# The Evolution and Expression of the Moth Visual Opsin Family

**DOI:** 10.1371/journal.pone.0078140

**Published:** 2013-10-30

**Authors:** Pengjun Xu, Bin Lu, Haijun Xiao, Xiaowei Fu, Robert W. Murphy, Kongming Wu

**Affiliations:** 1 State Key Laboratory for Biology of Plant Diseases and Insect Pests, Institute of Plant Protection, Chinese Academy of Agricultural Sciences, Beijing, P.R. China; 2 Department of Herpetology, Chengdu Institute of Biology, Chinese Academy of Sciences, Chengdu, Sichuan, China; 3 State Key Laboratory of Genetic Resources and Evolution, Kunming Institute of Zoology, Chinese Academy of Sciences, Kunming, P.R. China; University of Western Australia, Australia

## Abstract

Because visual genes likely evolved in response to their ambient photic environment, the dichotomy between closely related nocturnal moths and diurnal butterflies forms an ideal basis for investigating their evolution. To investigate whether the visual genes of moths are associated with nocturnal dim-light environments or not, we cloned long-wavelength (R), blue (B) and ultraviolet (UV) opsin genes from 12 species of wild-captured moths and examined their evolutionary functions. Strong purifying selection appeared to constrain the functions of the genes. Dark-treatment altered the levels of mRNA expression in *Helicoverpa armigera* such that R and UV opsins were up-regulated after dark-treatment, the latter faster than the former. In contrast, B opsins were not significantly up-regulated. Diel changes of opsin mRNA levels in both wild-captured and lab-reared individuals showed no significant fluctuation within the same group. However, the former group had significantly elevated levels of expression compared with the latter. Consequently, environmental conditions appeared to affect the patterns of expression. These findings and the proportional expression of opsins suggested that moths potentially possessed color vision and the visual system played a more important role in the ecology of moths than previously appreciated. This aspect did not differ much from that of diurnal butterflies.

## Introduction

The survival of species often involves adaptation to their environment. Vision is one of the most important senses of organisms and selection to the ambient light-environment may drive its evolution. The adaptive evolution between the visual systems of organisms and their ambient light-environment forms a model system for evolutionary research. For example, the opsins and eye morphology of fish are consistent with a possible adaptation to their ambient light environment [1]–[6].

Visual perception and the associated structural components are highly developed in insects. Lepidopterans, both butterflies and moths, depend on visual perception for foraging [7], [8], host plant identification [9], mate-choice [10] and long-distance migration [11]. Butterflies have evolved apposition eyes to better adapt their diurnal lifestyle, moreover, the opsin genes also adaptively evolved in color vision [12]–[15]. Unlike diurnal butterflies, most moths are primarily nocturnal. As with butterflies, they also require vision for feeding and long-distant migration [7], [16]–[18]. The superposition eyes of moths enhance light-capture and this may reflect an adaptation to dim-light vision [12]. Notwithstanding, little is known about how nocturnal lifestyle may have driven the evolution of opsin genes in moths.

Opsins are G-protein-coupled receptors characterized by seven transmembrane domain structures. A lysine residue in the seventh helix of opsins binds a light-sensitive vitamin A-derived chromophore and they determine the spectral sensitivity of the photopigments together [19]. Physiological and phylogenetic analyses indicate that early insects possessed trichromatic vision based on spectral peaks of pigments and subfamilies of opsins: long-wavelength (R) sensitive (>500 nm) proteins, blue (B) sensitive (400–500 nm) proteins and ultraviolet (UV) sensitive (325–400 nm) proteins [20], [21]. Although some insects retain trichromatic vision, many species appear to have adapted their range of spectral sensitivity to their ambient light environment. Gene duplications, losses and sequence variation of opsins occur, for example, in butterflies, mosquitoes and species of *Drosophila* [14], [15], [21]–[27]. Some members of Coleoptera lost B opsin [28], [29].

The dichotomy between closely related nocturnal moths and diurnal butterflies forms an ideal basis for investigating the evolution of visual genes in response to their ambient photic environment. We investigate the evolution of visual genes associated with nocturnal, dim-light environments by cloning 35 full-length cDNA opsin genes from 12 species of wild-captured moths. We analyze these data in concert with opsins from butterflies to investigate the evolutionary history of opsin and identify selection pressure genes in moths. We also seek to compare the patterns of diel-expression between wild-captured and lab-reared individuals of *Helicoverpa armigera*.

## Materials and Methods

### 1. Ethics Statement

With permission, we captured the insects in Changdao Experiment Station (Shandong) of Institute of Plant Protection, Chinese Academy of Agricultural Sciences. The wild-captured moths used in this study were serious pests in China, therefore, no permits were required for the described insect collection and experimentation.

### 2. Insects

Opsin genes were cloned from 12 species of moths, including *Agrotis segetum, Agrotis ypsilon, Argyrogramma agnate, Chilo supressalis, H. armigera, Loxostege sticticalis, Macroglossum stellatarum, Mamestra brassicae, Mythimna separata, Plutella xylostella, Spodoptera exigua, Spodoptera litura*. Specimens species were captured and stored in liquid N_2_ in July 2011 using a vertical pointing trap set up on Beihuang Island, Shandong province. This island, located in the Bohai Gulf (38° 23.200′ N, 120° 54,500′ E), had an area of about 2.5 km^2^ [16]–[18].

We chose *H. armigera* to investigate the expression patterns of opsins. Diel changes of mRNA levels under natural light in July 2011 were investigated in adult moths captured by light trap every 3 h beginning at 6∶00 (Beijing time). Moths were directly dropped into liquid N_2_. A lab-reared population from Langfang (Hebei province, 2005) was maintained at 25°C with a 14∶10, light:dark photoperiod. Different day-instar stages of adults, reared with a diet of 10% sugar and 2% vitamin complex, were collected daily at 9∶00. Diel changes of opsins mRNA level in the lab-reared population were determined from three day old adults (adults from the 3rd day after eclosion). Moths reared in a cage located at a window to sense the gradual change of light through dusk and dawn were collected every 3 h beginning at 6∶00 (Beijing time). The effect of dark-treatment on the expression pattern of opsins was investigated using moths three day old adults maintained in complete darkness and the control individuals reared at the same place while no dark-treatment.

### 3. Cloning and Sequencing of Opsins

Total RNA was isolated from individual adult moths using TRIzol reagent (Invitrogen, Carlsbad, CA, USA). Single stranded cDNA was synthesized using oligo(dT) and M-MLV Reverse Transcriptase (Promega, Madison, WL, USA). Genomic DNA was extracted from each individual using Easy Pure Genomic DNA Extraction Kit (TransGen, Beijing, China). The qualities of DNA, RNA and cDNA templates were ascertained by using conserved primers listed in Table S1 with the following PCR program: 4 min at 94°C; 30 s at 94°C, 30 s at 55°C, and 30 s at 72°C, 40 cycles; 10 min at 72°C.

Degenerate primers designed according to the known genes of other insects were used to amplify the 5′ends of opsins (Table S1), using the following PCR program: 4 min at 94°C; 30 s at 94°C, 30 s at 55°C, and 60 s at 72°C, 40 cycles; 10 min at 72°C. To obtain complete sequences of opsin, 5′ and 3′ RACE (Rapid Amplification of cDNA Ends) were performed using a SMART RACE cDNA Amplification Kit (Clontech, Palo Alto, CA, USA) with gene-specific primers according to the manufacture’s instructions (Table S1). To ensure the 5′/3′fragments were from the same gene, specific primers containing the full ORFs (open reading frames) were designed according to the 5′/3′ sequences of untranslated regions. In turn, these primers were used to amplify the entire ORF sequences with *pfu* enzyme (Table S1). The PCR program was as follows: 4 min at 94°C; 30 s at 94°C, 30 s at 55°C, and 150 s at 72°C, 40 cycles; 10 min at 72°C. All PCR products were purified, cloned into the pEASY-T Cloning Vector (TransGen, Beijing, China) and then used for the transformation of *Escherichia coli* DH5α. The clones were then sequenced.

### 4. Sequence Alignment, Phylogenetic and Evolutionary Analyses

All nucleotide sequences, including our sequences and others obtained from GenBank (Table S2), were aligned using Clustal W implemented in BioEdit [30] and then manually adjusted. The codon-matched sequences were phylogenetically analyzed using maximum likelihood (ML) and Bayesian inference (BI) with the same outgroup for each gene. ML analyses were performed using RAxML 7.3.2 [31] under the GTR+G substitution model. For estimating nodal support, nonparametric bootstrap proportions [32] with 1000 pseudoreplicates were used. BI analyses were performed using MrBayes 3.1.2 [33], [34]. For each gene, we partitioned the dataset according to codon position in each gene. The multiple partitions controlled for heterogeneity across dataset, such as variation in substitution rates. The best-fitting substitution-model for each partition (Table 1) was selected using JMODELTEST 0.1.1 [35]. The Bayesian information criterion (BIC) was used to select a model because of its high accuracy and precision [36]. For each partition-strategy, Metropolis-coupled Monte Carlo Markov chains were run for 10 million generations with two parallel searches using one cold and three heated chains, each starting with a random tree. Trees were sampled every 1000 generations using split frequencies <0.01 to indicate convergence. TRACER 1.5 [37] was used to determine when the log likelihood (lnL) of sampled trees reached a stationary. In all BI analyses, apparent stationarity was reached within 1 million generations; we conservatively discarded the first 5 million generations from each run as “burn-in” and used the sampled trees from the remaining 5 million generations (5001 trees) to calculate the frequency of nodal resolution in a 50% majority-rule consensus tree, termed Bayesian posterior probabilities (BPPs). Three replicate analyses were conducted to assess whether individual runs failed to converge upon the optimal posterior distribution and if likelihood values, branch lengths, tree topology, and posterior probabilities differed between runs. ML bootstrap proportions (MLBs) ≥70% and BPPs ≥0.95 were considered to indicate strong support for individual nodes [33], [38], [39]. The approach potentially assessed discordance among trees based on the implemented method of inference and provided a preferred topology for selection estimates.

**Table 1:** Model selection for each codon position of genes in MrBayes analysis.

Partitions	Best model	Gamma	Proportion of invariable sites
R_1_st_	K80+G	0.2680	–
R_2_nd_	GTR+G	0.2130	–
R_3_rd_	GTR+I+G	3.6510	0.0700
B_1_st_	SYM+I+G	0.8000	0.4100
B_2_nd_	K80+G	0.2130	–
B_3_rd_	GTR+G	1.2340	–
UV_1_st_	GTR+I+G	2.5690	0.5790
UV_2_nd_	K80+I+G	0.6620	0.5900
UV_3_rd_	HKY+I+G	3.4810	0.0550

### 5. Branch and Branch-Sites Test of Selection

Maximum Likelihood [40] was employed to test for differences in selection pressure, using the CODEML program of PAML 4.5 [41]. Branch models and branch-site models were employed to detect positive selection acting on the particular lineages. These tree-based tests of selection required the unrooted input-trees. RAxML and Mrbayes trees were converted to unrooted trees by introducing opsin genes of *Drosophila_melanogaster*. To test the hypothesis, for branch models, five hypotheses were evaluated: 1) one *d_n_/d_s_* ratio for all branches; 2) *d_n_/d_s_* ratio = 1 for all branches; 3) moth lineage and butterfly lineage have different *d_n_/d_s_* ratio (ω_2_ and ω_1_); 4) neutral evolution for moth (ω_2_ = 1); 5) the free-ratio model with free *d_n_/d_s_* ratio for each branch. For branch-site modes, the moth lineage was defined as the foreground branch, and remaining lineages were defined as background branches, which were specified in the tree file by using branch labels. Likelihood ratio test (LRT) was used to investigate if the alternative model, indicating positive selection, was superior to the null model.

### 6. Real-time PCR Analysis

Total RNA was isolated and cDNA was synthesized from whole individual adults. Using *β-actin* as the reference gene, real-time PCR (qPCR) was carried out with the TaqMan method (FAM) in 20 µl reaction agent comprised of 1 µl of template cDNA, 2*Premix Ex Taq™ (Takara, Japan), 0.2 µM of each primer and 0.4 µM probe (Table S1) on a 7500 Fast Real-time PCR System (Applied Biosystems). Thermal cycling conditions were: 95°C, 2 min and 45 cycles of 95°C for 15 s, 60°C for 34 s. After PCR, a melting curve analysis was performed to demonstrate the specificity of the PCR product, as displayed by a single peak. The cDNA sample of each group was replicated three times. Fold differences of the genes were calculated according to the 2^−ΔΔCT^ method [42]. Four repeats were used for each data-point.

For comparing proportional changes of mRNA levels of opsins, the absolute quantification methodology using standard curve was performed [43]. Fragments containing the primers and probes of qPCR of opsin genes were amplified with our de novo primers (Table S1) and cloned into the pEASY-T Cloning Vector (TransGen, Beijing, China). These plasmids were used for quantification assay.

Statistical analyses were conducted using STATA v.9.0. Student’s t-test or ANOVA with Bonferroni multiple comparisons were used to determine the level of significance in the relative levels of mRNA expression.

## Results

### 1. Moth Opsins

Highly conserved regions of aligned lepidopteran opsin sequences occurred in the 5′ untranslated region (UTR) of R, B and UV opsins, respectively (figure S1). After locating the ORFs of R, B and UV opsins, pairs of primers were designed based on conserved regions and there were used to amplify the 5′ end fragments of moth opsin genes containing the start codon. Taken together with the 3′ RACE, we successfully obtained complete full-length cDNA of 35 opsins from 12 species of moths. A Blast search at NCBI (http://www.ncbi.nih.gov/BLAST/) revealed that these genes were highly similar to opsins of other insects, especially Lepidoptera. No pseudogenes were found. Alignment of deduced amino acid sequences showed that these genes exhibited typical opsin characteristics, such as seven transmembrane domains, two sites with disulphide cysteine, and a lysine residue that binds the retinal chromophore through a retinylidine Schiff-base linkage in the seventh transmembrane domain (figure S2).

### 2. Phylogenetic Inference

**Figure 1:**
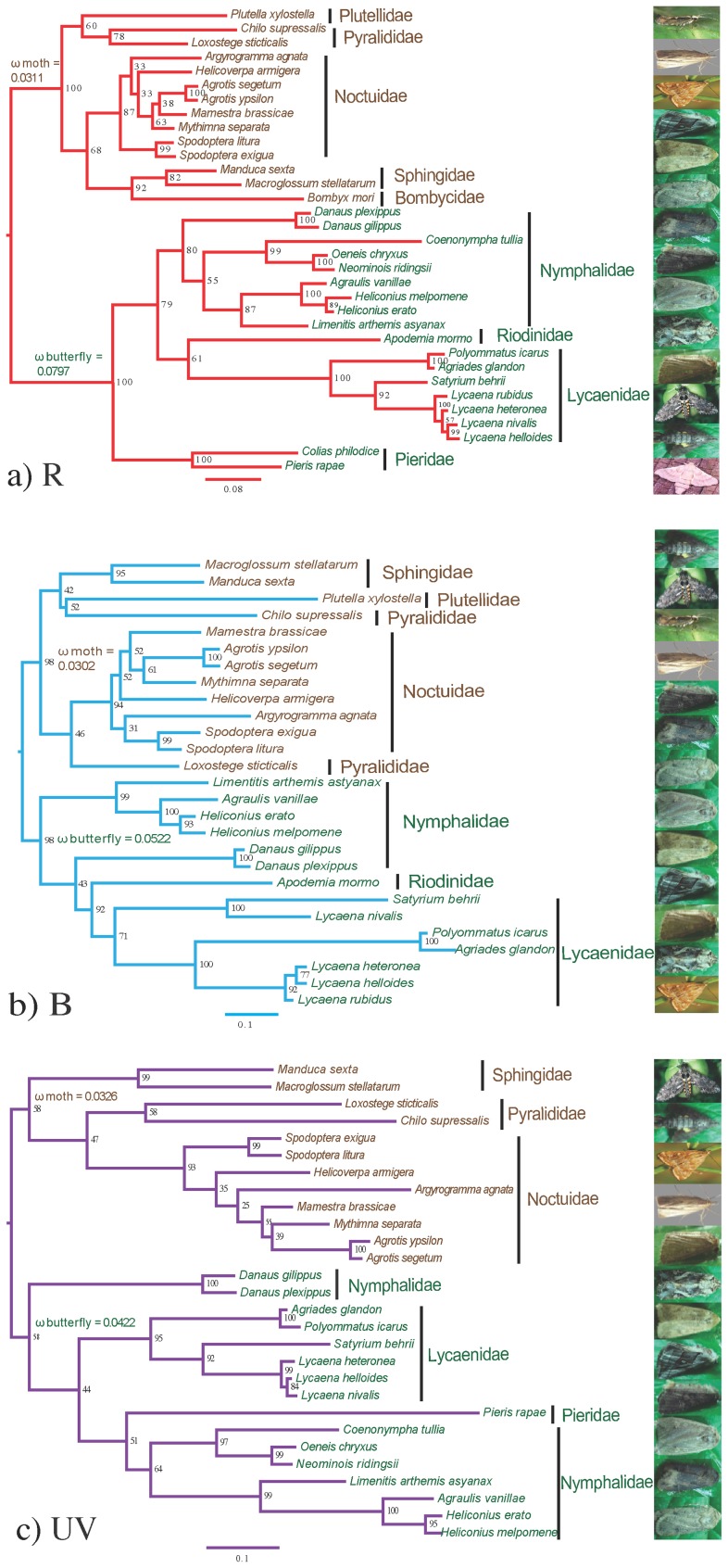
Phylogeny of opsin genes in Lepidoptera based on maximum likelihood. Values on the nodes are the nonparametric bootstrap proportions (MLBPs). Moths denoted in blue and butterflies in red. Branch-specific ω values are shown on nodes of the common ancestors. a) R opsin; b) B opsin; c) UV opsin. Figures at right show the moths.

ML and BI analysis generated quite different topologies depending on the gene and method of tree construction. One possible reason was the absence of sufficient information to resolve the phylogenetic relationships among moths using each gene alone. After concatenating all opsin genes, the topologies from ML and BI still differed. Consequently, both the ML (figure 1) and BI (figure S3) trees for each opsin genes were used for evaluating selection only; this study did not seek to resolve the phylogeny among these moths.

### 3. Natural Selection Analysis

The free-ratio model provided a better fit to these three opsin genes. Similar conditions occurred among the three opsin genes. For branch model, significant differences between moths and butterflies in *d_n_*/*d_s_* were not detected (Table 2). The branch-specific ω values of moths were 0.0311, 0.0302 and 0.0326 for R, B and UV opsins, respectively. The corresponding ω values of butterflies were higher than those of moths: 0.0797, 0.0522 and 0.0422, respectively (figure 1). Therefore, the hypothesis of strong purifying selection for moths was not rejected. For the branch-site model, the null model also could not be rejected. Similar results were generated for each gene regardless of employment of a ML or BI tree. All analyses indicated that the three types of opsin genes experienced strong purifying selection. The different topologies did not affect the selection-analyses. Therefore, we only showed the selection-analyses results using the ML tree.

**Table 2:** Selective patterns for opsin genes.

Genes	Model	np^a^	Ln L^b^	Estimates of ω	Models compared	LRT^c^	*P* Values
**R**	**Branch model**						
	I: one ratio	67	−12916.56	ω = 0.0561			
	J: one ratio ω = 1	66	−15114.28	ω = 1	J vs. I	4395.44	<0.001
	K: the moth branch has ω_2_, thebutterfly branch has ω_1_	69	−12874.53	ω_2_ = 0.0311, ω_1_ = 0.0797	I vs. K	84.06	<0.001
	L: the moth branch has ω_2_ = 1	68	−14028.98	ω_2_ = 1, ω_1_ = 0.0800	L vs. K	2308.90	<0.001
	M: each branch has its own ω	131	−12810.82	Variable ω by branch	I vs. M	211.48	<0.001
	**Branch-site models**						
	N: the moth branch	70	−12829.71				
	O: the moth branch has ω = 1	69	−12829.71		N vs. O	0	1
**B**	**Branch model**						
	A: one ratio	55	−12179.06	ω = 0.0397			
	B: one ratio ω = 1	54	−14629.01	ω = 1	B vs. A	4899.90	<0.001
	C: the moth branch has ω_2_, thebutterfly branch has ω_1_	57	−12164.44	ω_2_ = 0.0302, ω_1_ = 0.0522	A vs. C	29.24	1.0E-6
	D: the moth branch has ω_2_ = 1	56	−13444.93	ω_2_ = 1, ω_1_ = 0.0513	D vs. C	2560.98	<0.001
	E: each branch has its own ω	107	−12087.93	Variable ω by branch	A vs. E	182.26	<0.001
	**Branch-site models**						
	G: the moth branch	58	−12133.04				
	H: the moth branch has ω = 1	57	−12133.04		H vs. G	0	1
**UV**	**Branch model**						
	P: one ratio	57	−11648.23	ω = 0.0369			
	Q: one ratio ω = 1	56	−14141.76	ω = 1	Q vs. P	4987.06	<0.001
	R: the moth branch has ω_2_, thebutterfly branch has ω_1_	59	−11642.40	ω_2_ = 0.0326, ω_1_ = 0.0422,	P vs. R	11.66	0.0029
	S: the moth branch has ω_2_ = 1	58	−12807.41	ω_2_ = 1, ω_1_ = 0.04075	S vs. R	2330.02	<0.001
	T: each branch has its own ω	111	−11577.18	Variable ω by branch	P vs. T	142.10	<0.001
	**Branch-site models**						
	U: the moth branch	60	−11561.14				
	V: the moth branch has ω = 1	59	−11561.14		V vs. U	0	1

a Number of parameters.

b The natural logarithm of the likelihood value.

c Twice the log likelihood difference between the two models.

### 4. The Expression Level of Opsins mRNA

**Figure 2:**
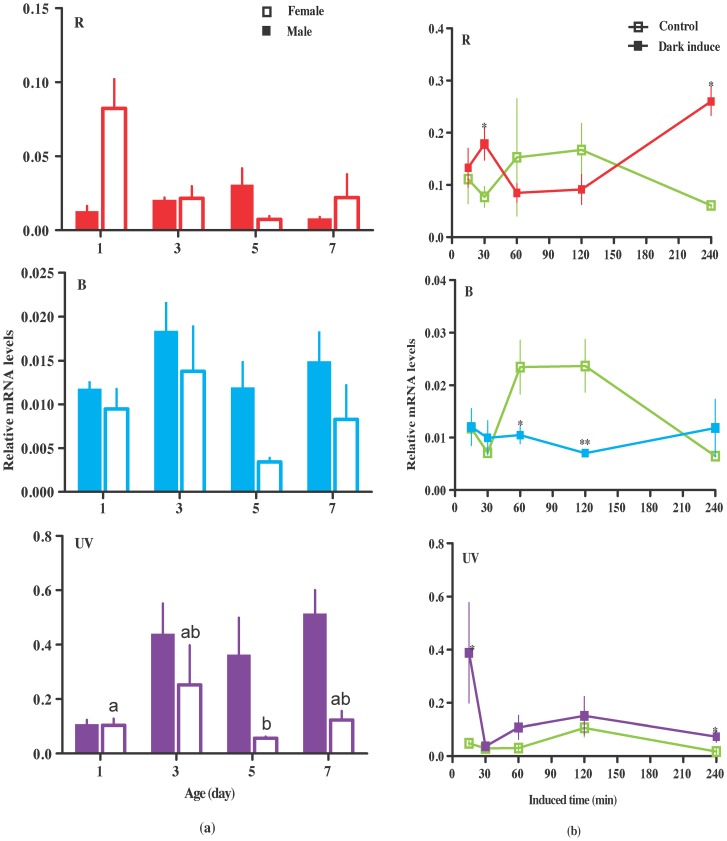
Relative expression level of R, B and UV opsins at different day instar stage of adults (a) and time of dark-treatment (b). Mean ± SE. n = 4 for each point. No significant difference occurs among different day instar stage of adults except for UV opsin in females, which is shown using different letters. The “*” and “**” denote statistical significance of the expression levels at *P*<0.05 and *P*<0.01, respectively.

Lab-reared *H. armigera* was used to investigate variation among different instar and dark-treated changes in opsin-expression. The results of qPCR indicated that the expression of opsins was dynamic and differed among day-instar-stage of adults but there were no significant difference among different days except for UV in female between day 1 and day 5 (Bonferroni multiple comparisons P = 0.033) (figure 2a). Dark-treatment changed the expression of opsins. The highest expression of UV opsin occurred at 15 min of darkness and then expression was down-regulated to a level similar to untreated individuals. R opsin was significantly up-regulated at 30 min then down-regulated at 1 h and 2 h followed significantly by the highest expression at 4 h. B opsin mRNA was significantly down-regulated after 1 h of darkness, and then up-regulated at 4 h albeit not significantly so (figure 2b).

**Figure 3:**
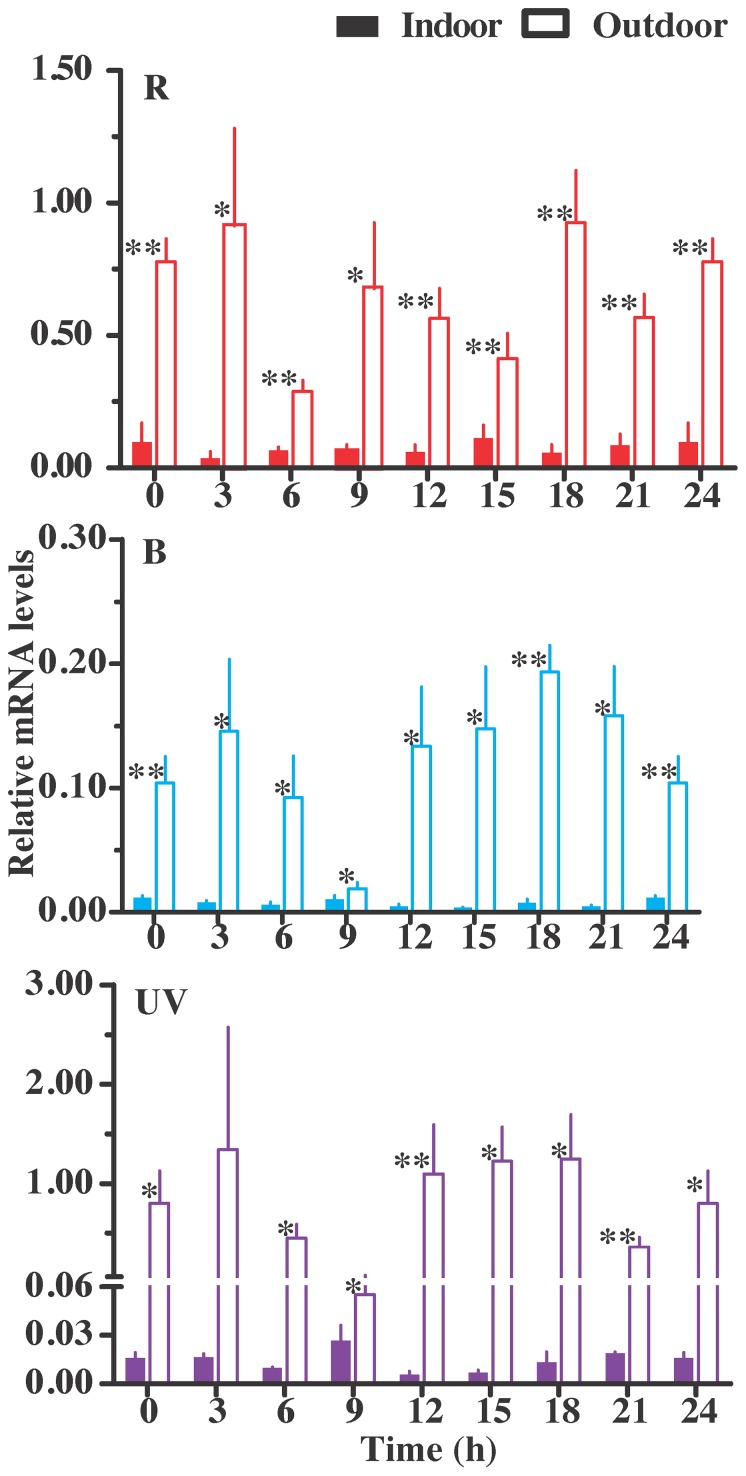
Relative expression level of R, B and UV opsins between indoor and outdoor *Helicoverpa armigera*. Mean ± SE. “Indoor” stands for lab-reared individuals, n = 4 for each point. “Outdoor” stands for the wild-captured individuals, n = 4 for each point except for UV at 3∶00 (n = 3) and 21∶00 (n = 2). The “*” and “**” denote statistical significance of the expression levels at *P*<0.05 and *P*<0.01, respectively.

**Figure 4:**
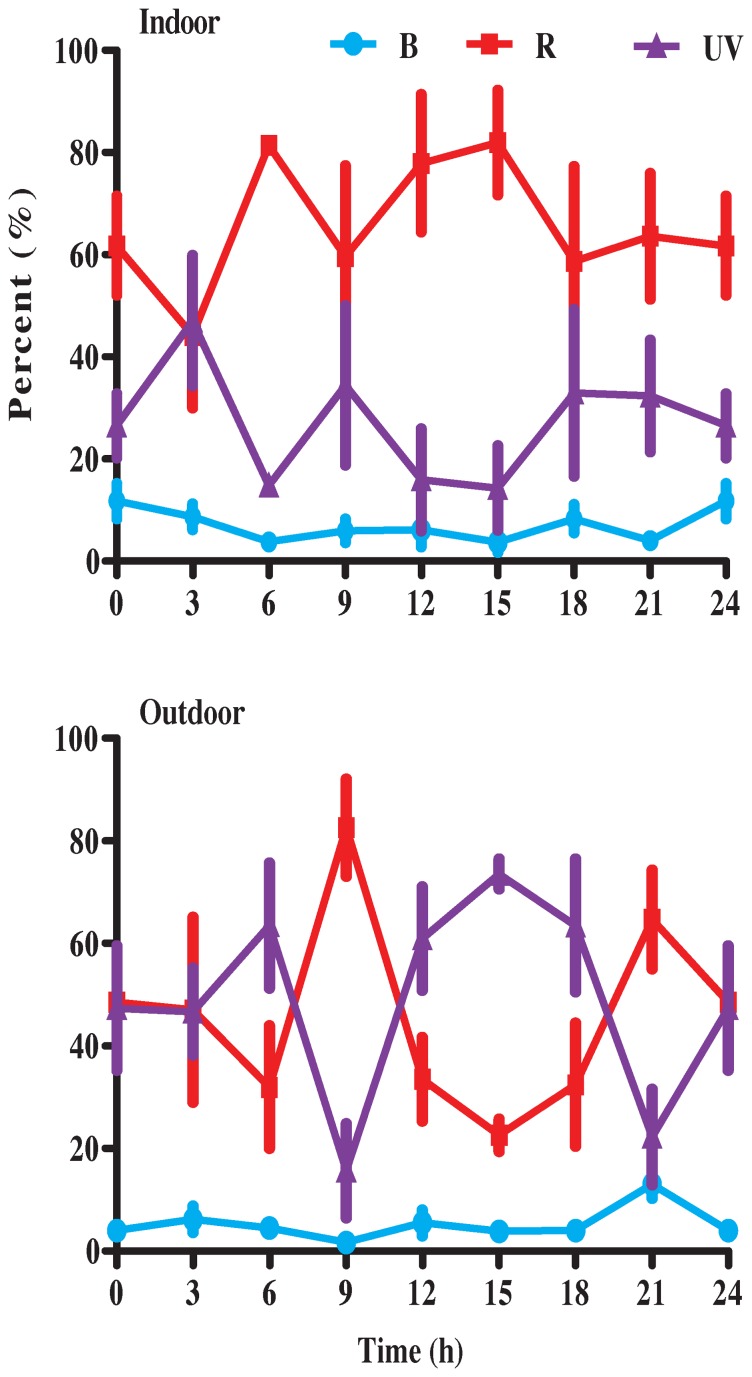
Proportional (expression relative to total opsin pool) expression of R, B and UV opsins in *H. armigera* including indoor and outdoor individuals. Mean ± SE.

The qPCR analysis did not reveal significant fluctuations of the three opsins within the same group (for wild-captured individuals, B: F = 1.90, d.f. = 7, 24, P = 0.1142; R: F = 1.50, d.f. = 7, 24, P = 0.2143; UV: F = 1.00, d.f. = 7, 21, P = 0.4596; for lab-reared individuals, B: F = 1.78, d.f. = 7, 24, P = 0.1377; R: F = 0.37, d.f. = 7, 24, P = 0.9082; UV: F = 2.32, d.f. = 7, 24, P = 0.0598). The wild-captured moths had significantly higher measures of relative opsin-expression than lab-reared moths at almost all time-points in *H. armigera* (figure 3, students t test P<0.05 and P<0.01, respectively). The standard curves for each opsin gene were constructed (figure S4) and these served as proportional measures of opsin-expression. The analysis indicated that the expression of UV opsins was proportionally higher in wild-captured than in lab-reared *H. armigera* (figure 4, figure S5). The proportional change of B opsin was stable in both wild-captured and lab-reared individuals (figure 4).

## Discussion

### 1. The Evolution of Moth Opsin

Selection pressure promotes the evolution of sensory systems by adapting organisms to local ecological conditions [44]. The cloning of opsins from several species of moths facilitates the identification of potential evolutionary adaptations of moths to their ambient light environments. The amino acid sequences of the moth pigments show a high percentage of identity with other arthropod opsins and possess a retinal binding site at site 296 (K296), a feature typical of all opsins. In vertebrate visual opsins, E113 acts as a counterion to stabilise the protonated Schiff base, however, in many vertebrate non-visual pigments and invertebrate opsins, the counterion has shifted to site 181 (E181) with a subsequent E113Y substitution [19]. In moths, Y113 is present in blue- and red-sensitive pigments, F113 in UV-sensitive opsins, and E181 in all three opsin classes. Strong signals of purifying selection exist in the three opsin genes. This suggests that these genes have conserved functions in moths. The rod-only retina of deep-sea fishes is sensitive to wavelengths of approximate 480 nm, which matches the ambient dim-light [45]–[48]. Surely, species living in the dim-light environment still need to capture available light. Therefore, opsin genes of species living in dim-light environments should retain functions.

Most species of moths are either nocturnal or crepuscular. Twilight is blue-shifted relative to daylight and may also contain a relatively strong red light [21]. Interestingly, our results show that B and R opsins of moths, which respond to blue and red light, are under strong purifying selection. In moths, these two genes seem to have functional constraints. Unlike moths, the B and R opsins of many diurnal butterflies are highly diverse. This likely reflects adaptations to variable light-niches [14], [22], [49]. However, the loss of B in the darkling beetle (*Tribolium castaneum*) may be an evolutionary sensory modality tradeoff, most likely driven by the dramatic increase of chemoreceptor rather than a nocturnal lifestyle [28].

UV opsin is the short-wavelength opsin that functions in color vision and strong UV light exposure, which might be harmful to nocturnal insects [50], [51]. Generally, UV opsin might not be so important to nocturnal moths because UV light is weakest at night. However, our results indicate that this gene continues to experience functional constraints. Two non-exclusive observations may explain the function of UV opsin in moths. First, moths have limited diurnal activities though they are far more active at night. In this scenario, UV opsin functions to protect them from UV damage. Second, in addition to offering protection from UV light, UV opsin may still play an important role in color vision, and perhaps other functions. In this case, we do not expect a functional shift in moths that differs from that of diurnal butterflies.

### 2. The Expression Pattern of Moth Opsin

Adaptation of the visual system usually involves two mechanisms: spectral tuning by amino acid sequence variation and expression-regulation [52]–[55]. The levels of opsin-expression could be a function of visual pigment sensitivity. In this case, regulation is a response to conditions such as light, age and heredity [2], [5], [56]–[59]. Our results show that the expression of opsins in *H. armigera* also changes after dark-treatment yet uniquely. UV and R opsins are immediately up-regulated, although the latter occurs more slowly than the former, In contrast, dark-treatment does not significantly up-regulate B opsin. Taken with the evidence that strong purifying selection acts on all three kinds of opsin genes, we infer that the opsin genes not only work well for moths, they also facilitate nocturnal activities. Electroretinogram (ERG) analyses of *H. armigera* suggest that photosensitivity gets stronger with dark-treatment ≤2 h, especially in the UV region [60]. Variation occurs in the significant up-regulation of R and UV opsins but not at each point of detection, yet in other cases significant up-regulation of B opsin is not detected within the first 2 h of dark-treatment. During this time, the screening pigments rapidly moved making the compound eye transform from being light in appearance to becoming black (data not shown). This observation suggests that the superposing optics and movement of screening pigments also play important roles in the nocturnal lifestyle of moths. Some nocturnal butterflies also evolved superposing optics [61] and this observation supports our conjecture.

The expression-patterns of opsin genes correspond significantly with environmental light [2], [4], [5], [59]. Phototaxis has been well-established in moths [62], [63]. However, no studies report diel changes of mRNA levels. Our comparison of wild-captured and lab-reared (>5 yr) *H. armigera* fails to detect significantly different levels of RNA between day and night within the same group, yet opsins are down-regulated significantly in lab-reared *H. armigera*. This observation most likely results from the weaker light in the laboratory as opposed to the wild. Consequently, the expression-patterns of moth opsins could vary in different environments. The relative measures of opsin-expression to the housekeeping gene may indicate differences in diel opsin-regulation both within and among species. However, proportional measures of opsin-expression might serve better for making inferences about color vision than relative measure [5]. Diurnal insects have elaborated optic systems that use ambient light for color-vision and navigation [11], [21]. Vision is involved in inter-individual communication. In contrast, nocturnal insects usually communicate using pheromones or songs [64]. Nocturnal insects also have the ability to use light for color vision and flight control [65]–[67]. Our proportional measures identify changes in expression in time in different test populations, suggesting that *H. armigera* has potentially nocturnal color vision. In particular, the proportion of UV opsin is lower in lab-reared than wild-captured moths, mostly due to small differences in exposure to UV light. This result indicates that UV opsin has an important function in wild moths.

## Conclusion

The three kinds of moth opsins are under strong purifying selection. The expression of opsin genes is plastic and varies with the environmental light. Relative and proportional measures of opsin-expression vary with time of day and environment, suggesting that the vision system plays a more important role in the sensory ecology of moths than previously appreciated.

## Supporting Information

Figure S1 **Alignments of 5′ untranslated region (UTR) of B (a), R (b) and UV (c) opsin genes from insects of Lepidoptera.** “.” indicates sites identical with the first row sequence. “−” signifies a gap. Highly conserved regions shown with shading.
(TIF)

Figure S2
**The alignments of amino acid sequences of B (a), UV (b) and R (c) opsins amplified in this study.** “.” indicates sites identical to the first row sequence. Transmembrane domains are shaded. 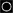 = the site of the chromophore Schiff-base linkage (K). • = the counterion (Glu) (E). ▾ = two disulphide Cys (C) residues. ▪ =  Position 90 in bovine rodopsin, the residues for blue and UV. ○ =  Position 113 in bovine rodopsin. AA =  *Argyrogramma agnata*, AS =  *Agrotis segetum*, AY =  *Agrotis ypsilon*, CS =  *Chilo supressalis*, HA =  *Helicoverpa armigera*, LS =  *Loxostege sticticalis*, MB =  *Mamestra brassicae*, MS =  *Mythimna separata*, MSt =  *Macroglossum stellatarum*, PX =  *Plutella xylostella*, SL =  *Spodoptera litura*, SE =  *Spodoptera exigua*.(TIF)

Figure S3 **Phylogenetic reconstruction of opsin genes in Lepidopteran based on Bayesian inference.** Values on the nodes are the Bayesian posterior probabilities (BPPs). Moths denoted in blue and butterflies in red. a) R opsin; b) B opsin; c) UV opsin.
(TIF)

Figure S4 **The standard curves for opsins of** ***H. armigera*** **determined by triplicate sampling.** The primer efficiency is 103.7%, 98.7% and 100.7% for R, B and UV opsins, respectively.
(TIF)

Figure S5 **Proportional (expression relative to total opsin pool) expression of UV opsins in** ***H. armigera*** **between indoor and outdoor individuals.** Mean ± SE. The “*” and “**” denote statistical significance of the expression levels at *P*<0.05 and *P*<0.01, respectively.
(TIF)

Table S1 **Primers used in this study.** The abbreviation of species name was shown in bracket.
(PDF)

Table S2 **The GenBank accession number of genes used in this study.**
(PDF)
