# Supplementary figures and images for: The Evolution and Expression of the Moth Visual Opsin Family
Source: PLoS One. 2013 Oct 30;8(10):e78140. doi: 10.1371/journal.pone.0078140 (PMC3813493; doi:10.1371/journal.pone.0078140)

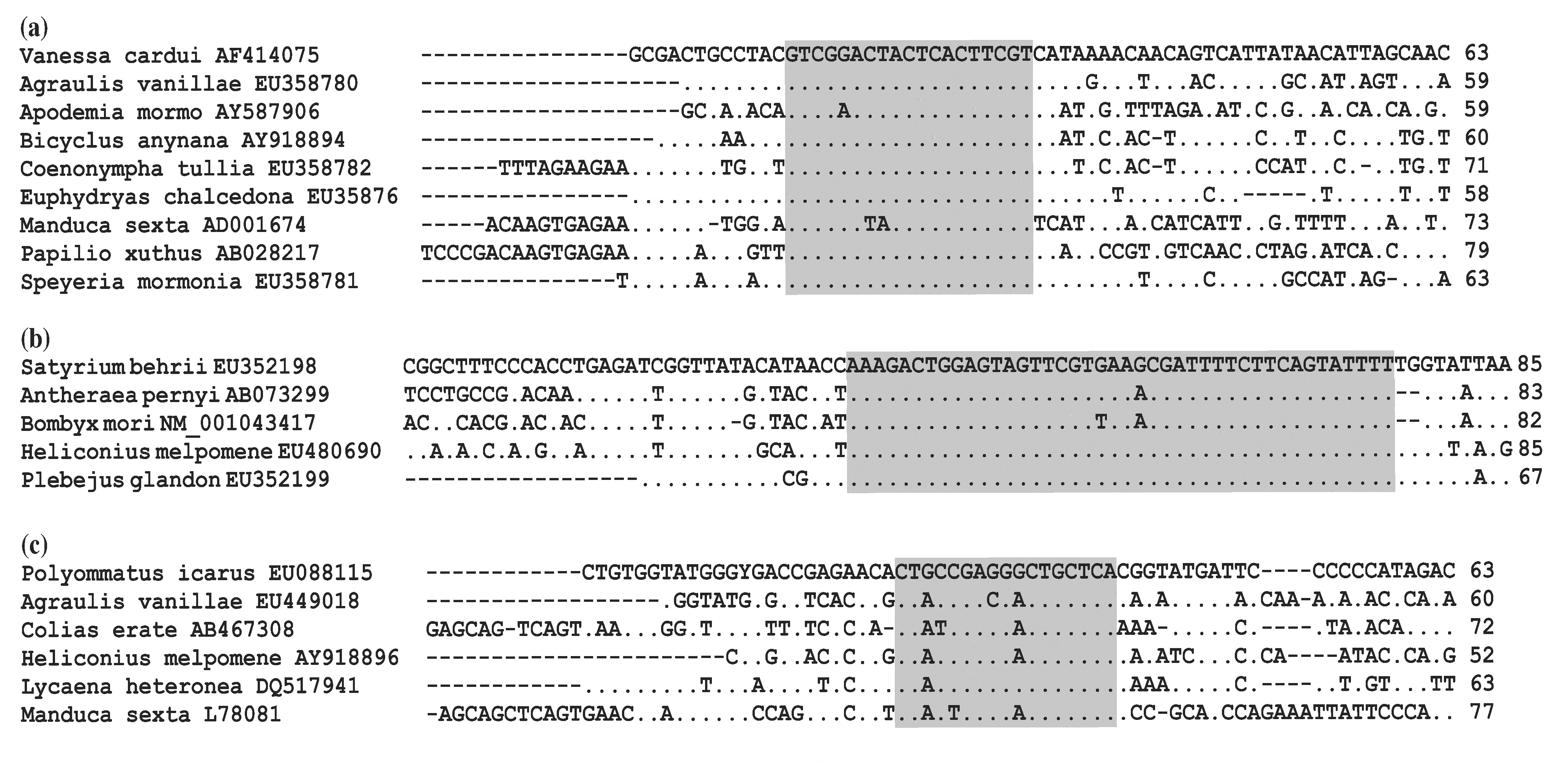

Supplement: Figure S1 — Alignments of 5′ untranslated region (UTR) of B (a), R (b) and UV (c) opsin genes from insects of Lepidoptera. “.” indicates sites identical with the first row sequence. “−” signifies a gap. Highly conserved regions shown with shading. (TIF) [file pone.0078140.s001.tif]

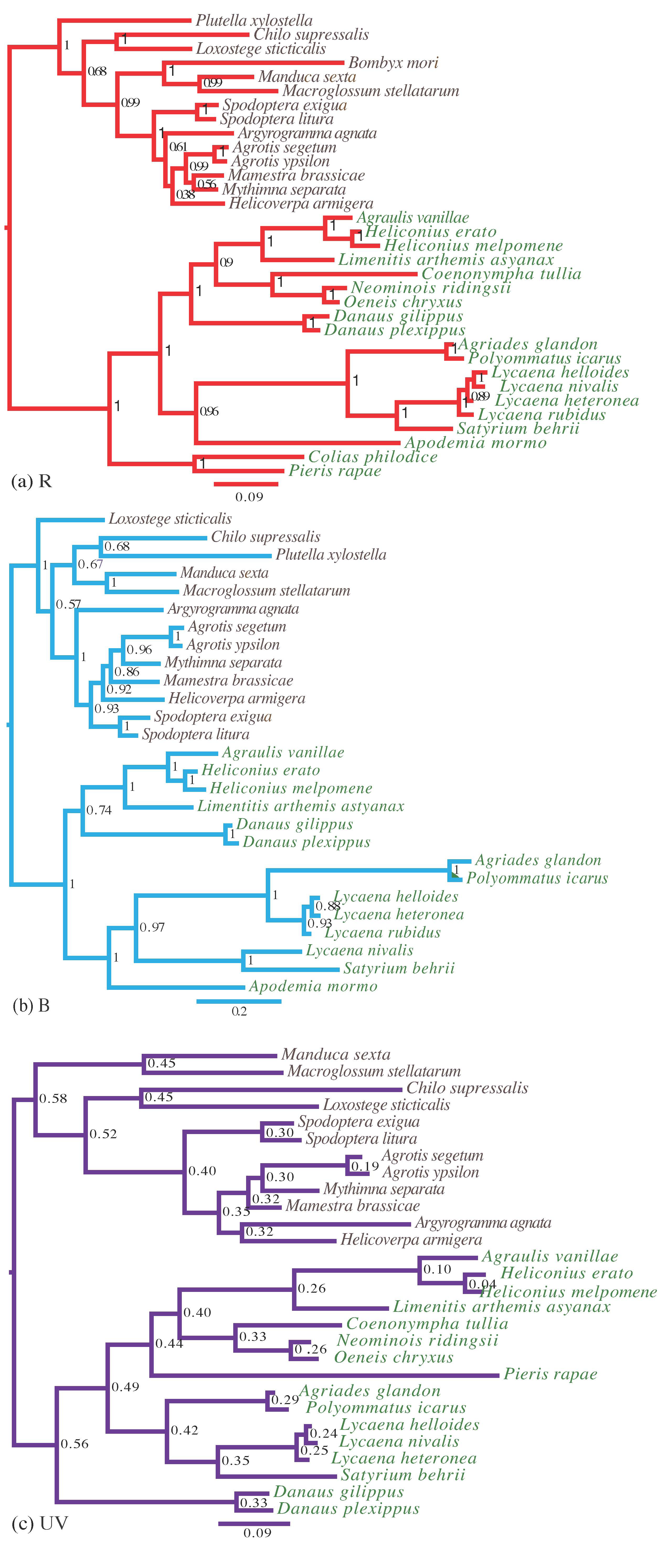

Supplement: Figure S3 — Phylogenetic reconstruction of opsin genes in Lepidopteran based on Bayesian inference. Values on the nodes are the Bayesian posterior probabilities (BPPs). Moths denoted in blue and butterflies in red. a) R opsin; b) B opsin; c) UV opsin. (TIF) [file pone.0078140.s003.tif]

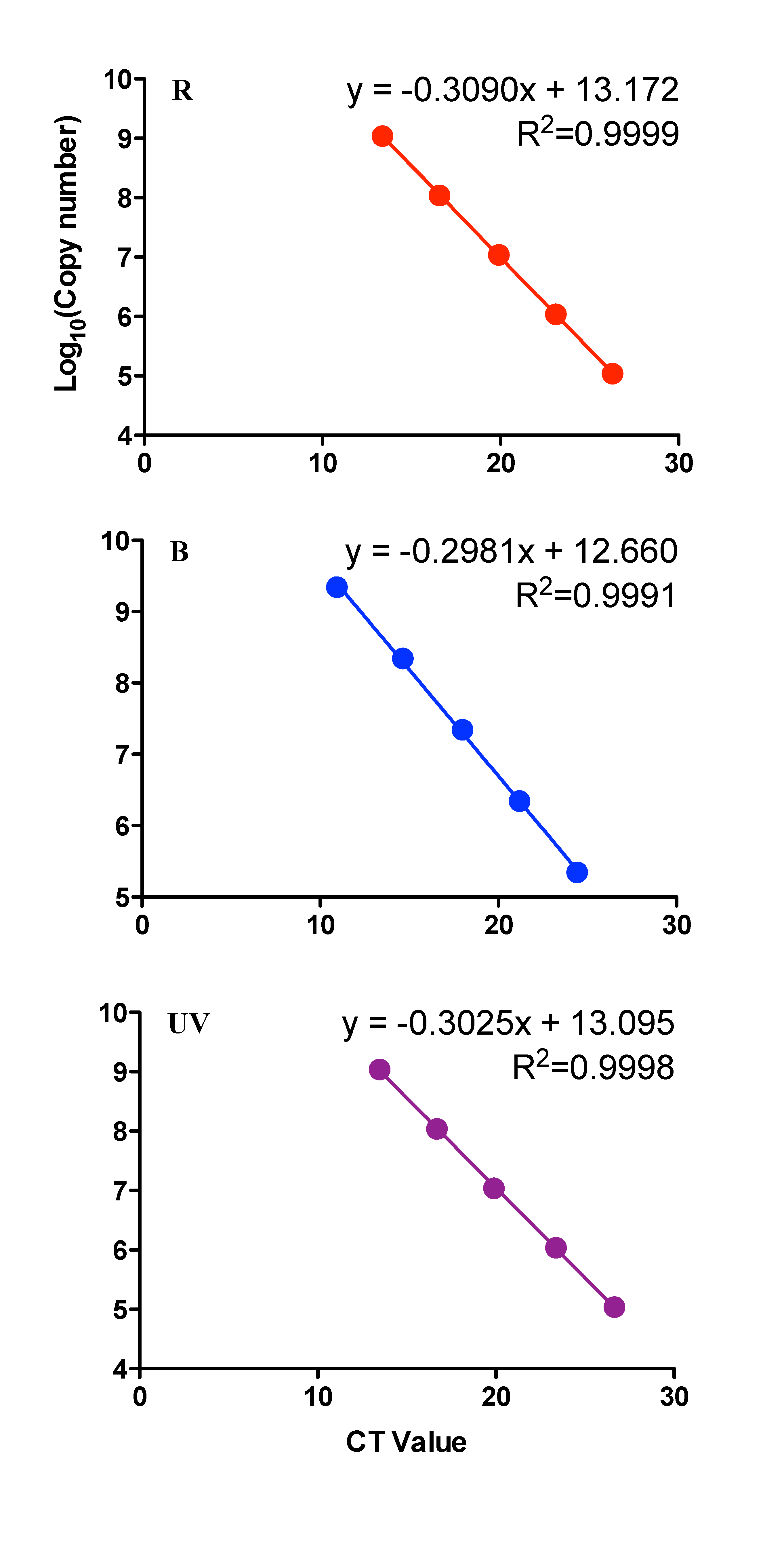

Supplement: Figure S4 — The standard curves for opsins of H. armigera determined by triplicate sampling. The primer efficiency is 103.7%, 98.7% and 100.7% for R, B and UV opsins, respectively. (TIF) [file pone.0078140.s004.tif]

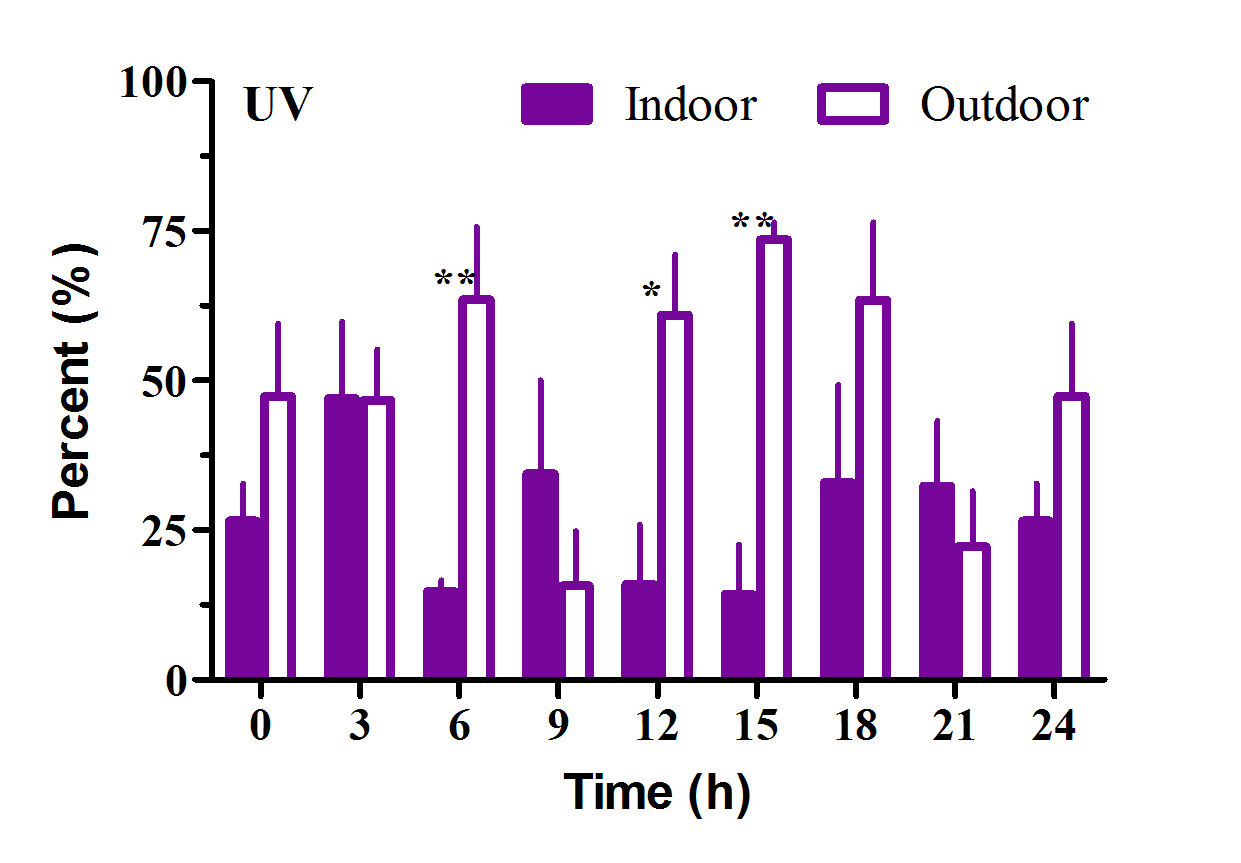

Supplement: Figure S5 — Proportional (expression relative to total opsin pool) expression of UV opsins in H. armigera between indoor and outdoor individuals. Mean ± SE. The “*” and “**” denote statistical significance of the expression levels at P<0.05 and P<0.01, respectively. (TIF) [file pone.0078140.s005.tif]
