# Supplementary material for: The Evolution and Expression of the Moth Visual Opsin Family
Source: PLoS One. 2013 Oct 30;8(10):e78140. doi: 10.1371/journal.pone.0078140 (PMC3813493; doi:10.1371/journal.pone.0078140)
Supplement: Table S1 — Primers used in this study. The abbreviation of species name was shown in bracket. (PDF) [file pone.0078140.s006.pdf]

Primer names	Primer sequence (5'-3')	Amplified fragments
B-F	GTCGRAYWACTCACTTCG	
B-R	CACRTTCATRTTCTTNGC	
LW-F	AAGACTGGAGTAGTTCG	Degenerate primers for the 5' ends of opsins
LW-R	TTVGCYTGYTCCCTCAT	
UV-F	CTRYYGASGRCTGCTCA	
UV-R	GCCCTCWGGWACRWATC	
AAB-3F1	ATGCCGCTGCTCATCACCAACTCCT	
AAB-3F2	ATCTCGTGCCCACTGGACGGTAGG	
AALW-3F1	ATGCTTGTGCTGGCTCTCTATTCGGTT	3' RACE for opsins from *Argyrogramma agnate* (AA)
AALW-3F2	GGTATCGCCGCCAAGCCAATGAC	
AAUV-3F1	TACCCGAGCACTGGCTGTCATACCC	
AAUV-3F2	TCGCCCTCTGGACGGTCGCTTATC	
FAAB-F	CATGAAACAATAGTTCAATCG	
FAAB-R	ATTGTTTCTATTGTATTGCGA	
FAALW-F	TTTTGTAATAACCATCTCGG	Full-length cDNA of opsins from *Argyrogramma agnate*
FAALW-R	CGAGTCGTAGAGATTGTGTC	
FAAUV-F	TTCTCCCAACATGTATAAC	
FAAUV-R	TTATTCCAGTCCATTATCG	
ASB-3F1	CACATCTTTCCTGGGCAACGGCAT	
ASB-3F2	GGCACTTCCATTCACCGTCCTTCC	
ASLW-3F1	ATGAACCCGCTTTGGCACGGACTT	3' RACE for opsins from *Agrotis segetum* (AS)
ASLW-3F2	TTGTGCGGGCTCGCTATTTGGATG	
ASUV-3F1	AGATGCTGGGCGATGGACTGACG	
ASUV-3F2	ATGCGAGGCTTTGCCAGTGGAACC	
FASB-F	ATCTTTATTCCATCTATCCCA	
FASB-R	CATTGTAGCAGGAGTTGTAGT	
FASLW-F	TAAGTGAACGACAACGACAGA	Full-length cDNA of opsins from *Agrotis segetum*
FASLW-R	AAATCGTGTGTCCATAGAGCA	
FASUV-F	CCTGCCCAACGTTCAATC	
FASUV-R	TTTTGGAGACGGCATCAG	
AYB-3F1	CACATCTTTCCTGGGCAACGGCAT	
AYB-3F2	GTTCTGGGCACTACCATTCACCGTCC	
AYLW-3F1	ATGGGGTCCTCTTGCGTGTGAACTCTAC	3' RACE for opsins from *Agrotis ypsilon* (AY)
AYLW-3F2	TACTCGGCATTTGGGCGTTCTCACTC	
AYUV-3F1	TGGACTGACGGGAGATGACCTGGC	
AYUV-3F2	TATGACAGGCATTCCACAATCACCCG	
FAYB-F	CTTTGTTCCATCTATCCCAT	
FAYB-R	CAGGAGTTGGGATTTCTG	
FAYLW-F	CGTTTAACGATTTTCTTCAG	Full-length cDNA of opsins from *Agrotis ypsilon*
FAYLW-R	TAAATAACATCGCTTTGGCA	
FAYUV-F	TGCCCAACGTTCAATCAAC	
FAYUV-R	ATCTTTTGGAGACAGCATCG	
CSB-3F1	CATTTACGCCATCCTGGGGTCGCT	
CSB-3F2	ACTTGGTTCTGGGCGTTACCGTTCA	
CSLW-3F1	GTTTCACTATCGGCGTCCTTGGCTTC	3' RACE for opsins from *Chilo supressalis* (CS)
CSLW-3F2	GCAAGGATTGGTTCAGCCGCAGTT	
CSUV-3F1	GGTTCGCAACAGGGACACTCGGC	
CSUV-3F2	GCTCTGCTGCCCCTCTTTGAAACTTG	
FCSB-F	ACGATAGTGTTTGTGACCG	

FCSB-R	TCAAGTCTTCGGTTCGTTA	
FCSLW-F	CCTTATCCTGGACCTTTTA	Full-length cDNA of opsins from *Chilo supressalis*
FCSLW-R	ATAACATCGCATTGGCAG	
FCSUV-F	ACTGAGCATAATGGAGAAC	
FCSUV-R	GATGGCTTCGTTTGTTAGA	
HAB-3F1	GCTGGGAAACGGCATAGTCATCTGGA	
HAB-3F2	CCAGGATACGAAGGTGTTTGTCGGTTG	
HALW-3F1	CCTCTCGCTTGTGAACTTTACGCTTGC	3' RACE for opsins from *Helivoverpa armigera* (HA)
HALW-3F2	ATCGTAAAAGGTATCGCCGCCAAGC	
HAUV-3F1	CGGGGCTGACGGGAGAAGACCTG	
HAUV-3F2	TGGGCTCTGCTGCCACTCTTCAAAATC	
FHAB-F	ATGGCGTCGAATTACTCAG	
FHAB-R	GTCTCTAAGCTTCAGCAGC	
FHALW-F	GTTTCACGATTTTCTTCAGT	Full-length cDNA of opsins from *Helivoverpa armigera*
FHALW-R	TAGTCCAAGATACAGAGCCT	
FHAUV-F	GCTGCTCATTCCTTCCCTAC	
FHAUV-R	TAAACTTGCCGTAGTCGTCG	
LSB-3F1	TAGCACATCCAAATCCCTCCGAAGC	
LSB-3F2	ACGGAAGGATAAACAAGGTCCAAGCG	
LSLW-3F1	CTTGGGTTTCACAATCGGCGTCCT	3' RACE for opsins from *Loxostege sticticalis* (LS)
LSLW-3F2	GCCAAGCCAATGACCAACAACGG	
LSUV-3F1	CGCACACAGCATTGGCTCTACTCTACAT	
LSUV-3F2	TCTTCATTTACAATAGTGCGATGCGAGG	
FLSB-F	TTTGAAAAAGCACACGCAC	
FLSB-R	CATCCAGTCATTGTCAAGC	
FLSLW-F	CAAATAGAAGTCGTGTAACG	Full-length cDNA of opsins from *Loxostege sticticalis*
FLSLW-R	ATAAATAACATCGCTTTGGC	
FLSUV-F	ACCAACCCAATCCATCATC	
FLSUV-R	AAGCAATCTTATGTTGAGGT	
MBB-3F1	CCGCTGGACGGAAGGGTGAACAA	
MBB-3F2	TTCGTTCCAGAGGGCTTCTTGACCAC	
MBLW-3F1	TGGGGTCCTCTTGCTTGCGAACTT	3' RACE for opsins from *Mamestra brassicae* (MB)
MBLW-3F2	AGCCGCCAAGCCAATGACTAACAAC	
MBUV-3F1	CAGCCGCTTTAGTGGGCAATGGA	
MBUV-3F2	GGACCTTCGGCTGCCAACTATTCG	
FMBB-F	TTCTACCTAACCCAAGATACA	
FMBB-R	GATACGATGTGTTGATGACG	
FMBLW-F	TTTTGTAATAACCATCTCGG	Full-length cDNA of opsins from *Mamestra brassicae*
FMBLW-R	AATCGTGTCCATTGAGTAGC	
FMBUV-F	TCCCAACATTCATTCAAC	
FMBUV-R	TAAAAAATGATAGGTCGTGC	
MSBO3F1	TTGTCATCTGGATTTTCGGCACCTCA	
MSBO3F2	ATTTGTTCCCGAGGGCTTCCTGACC	
MSLO3F1	GTGAAAGGTATCGCCGCCAAGCC	3' RACE for opsins from *Mythimna separate* (MS)
MSLO3F2	CGCATACTCGGCATCTGGGTGTTCTC	
MSUO3F1	GCAGCCGCTTTAGTGGGCAATGG	
MSUO3F2	ACCGTGGGCTCTACTGCCGCTCT	
FMSBO-F	TAAAACCTTTGTTCCATTCA	
FMSBO-R	CTCAAATCAACTGTCACTGT	
FMSLO-F	AAGTGAAGACAACAACAGCG	Full-length cDNA of opsins from *Mythimna separate*

FMSLO-R	TGTCCATAGAGTAGCGAGGC	
FMSUO-F	TCACTCCTTCCCAACATTC	
FMSUO-R	GTAGAGGACGGTAGGCAGT	
MStB-3F1	ATCTATGGCGTGTTCGGCTCCCTG	
MStB-3F2	CCCGAGGGTTTCCTGACGACTTGC	
MStLW-3F1	TCTTGGAATCTGGCTCTTCTCACTCGC	3' RACE for opsins from *Macroglossum stellatarum* (MSt)
MStLW-3F2	TGACTGCCTGCGGAACCGACTACTT	
MStUV-3F1	CCGTGGAGATGCTCGGTGATGGTT	
MStUV-3F2	CGCCACACCTTGGTCTCTTCTTCCTC	
FMStB-F	GTCGACCATTTTGTAAGC	
FMStB-R	CTTCAATGTTATGATGATTTC	
FMStLW-F	GGACTTTTTGAGTTAATTGC	Full-length cDNA of opsins from *Macroglossum stellatarum*
FMStLW-R	AGTGTCCTGCAATGTCG	
FMStUV-F	CGTAACCTGCAGAACTATC	
FMStUV-R	GGTAGTCCATCAGTAAAGCC	
PXB-3F1	CGGCGGTCAGCAAGTTCTGGCAC	
PXB-3F2	CGTTGGGGTCGCTGTCTGGGATAG	3' RACE for opsins from *Plutella xylostella* (PX)
PXLW-3F1	TGTGCTGGCTCTCTCTTTGGTTGCG	
PXLW-3F2	CCTGGGCATCTGGCTGTTCTCGC	
FPXB-F	AAAAAGCAACGGTTTTCAC	
FPXB-R	AATTTGAACTAAGCCTCCG	Full-length cDNA of opsins from *Plutella xylostella*
FPXLW-F	ATCTCGTAGTCCCCCTTAC	
FPXLW-R	GAGGTGATGTCTGGTGGAG	
SEB-3F1	CGGACAACTCCACAGACTTCGGCATT	
SEB-3F2	TCCTCATCGCATTCACCTGGTTCTGG	
SELW-3F1	CGCTATGGCACGGACTCTTGGGAT	3' RACE for opsins from *Spodoptera exigua* (SE)
SELW-3F2	GGTATGGGGTCCTCTTGCGTGTGAAC	
SEUV-3F1	GGAGCCCGAGTGAGGTGGAGATGC	
SEUV-3F2	CCCTTAGACGGGCGACTGTCACG	
FSEB-F	ACGCGCACAAGATGGCG	
FSEB-R	AGGGTCTAAGCTTCAGCCG	
FSELW-F	TTTTGTAATAACCATCTCGGA	Full-length cDNA of opsins from *Spodoptera exigua*
FSELW-R	AGTTTGTCATCGGAGGAGTC	
FSEUV-F	GCTCACTCCTTCAGTATTC	
FSEUV-R	GAAAAAGAGGCAAGTGAGC	
SLBO3F1	ACATCAAAATCCCTGCGAAGCCCA	
SLBO3F2	GTCCTCGGTTCCTTGTCGGGTATTG	
SLLO3F1	GTGGGTATGGGGTCCTCTTGCGTG	3' RACE for opsins from *Spodoptera litura* (SL)
SLLO3F2	CATCCTCGGCATCTGGGCATTCTC	
SLUO3F1	CGCAGGACTGACGGGGGAAGACC	
SLUO3F2	CTGGCTCTCGTTCCCCGCACCTC	
FSLBO-F	GAAAATCTATTACTTTATCCGC	
FSLBO-R	TGAGTGACAGATAAGATAGCCA	
FSLLO-F	AGATAAGAACACCACAACGA	Full-length cDNA of opsins from *Spodoptera litura*
FSLLO-R	TCGTGGAGGTTTGTTATCG	
FSLUO-F	TCCTTTAGTACCTATTCCATC	
FSLUO-R	CCATCAACACGATAAAACG	
Actin-F	CATCTACGAGGGTTACGC	Primers for detection of quality of RNA samples and cDNA templates
Actin-R	CATCTGTTGGAAGGTGGA	
HAB-QF	CTTCGACCTGATGATGATG	Primers of qPCR for B-opsin from *Helicoverpa*

HAB-QR	AACGCAATAACAGCATTG	*armigera*
HAprobe-B	TGCCGCTACTCGTCATCAACT	
HALW-QF	CTTGCTAGTGGTGAATCTTG	Primers of qPCR for LW-opsin from *Helicoverpa armigera*
HALW-QR	TACCCATGTTTCGTTGTAAC	
HAprobe-LW	TCTCCGACTTCCTGATGATGTGC	
HAUV-QF	CGTGTGCTTCTTATTTGTAG	Primers of qPCR for UV-opsin from *Helicoverpa armigera*
HAUV-QR	GGTGTTAATAGTTGTTGGTTTC	
HAprobe-UV	CGCCATAACGCCATAAGGTGTC	
HAactin-QF	CTGGGACGATATGGAGAA	
HAactin-QR	CGAACATGATCTGTGTCA	Primers of qPCR for actin from *Helicoverpa armigera*
HAprobe-actin	CACCACACCTTCTACAACGAGC	
Ha-B-QF	CTGGGAAACGGCATAGTC	
Ha-B-QR	CATCCTCCTTGTTGGCAG	Primers to construct plasmid for standard curve using in qPCR from *Helicoverpa armigera*
Ha-LW-QF	GGGATTCACTATCGGCTG	
Ha-LW-QR	CCTCATCCCCTTCTCGTG	
Ha-UV-QF	GCTCTGCTGCCACTGTTC	
Ha-UV-QR	GCTGTATCATCAGGCTCC	
