# Supplementary material for: The Evolution and Expression of the Moth Visual Opsin Family
Source: PLoS One. 2013 Oct 30;8(10):e78140. doi: 10.1371/journal.pone.0078140 (PMC3813493; doi:10.1371/journal.pone.0078140)
Supplement: Table S2 — The GenBank accession number of genes used in this study. (PDF) [file pone.0078140.s007.pdf]

Names of species	Blue opsin	Long-wavelength opsin	UV opsin
Agraulis vanillae	EU358780	DQ924367	DQ924367
Agriades glandon	DQ402503	EU352199	EU449024
Apodemia mormo	AY587906	AY587907	
Coenonympha tullia		DQ924374	DQ924374
Colias philodice		AY918900	
Danaus gilippus	EU358779	EU352197	EU449017
Danaus plexippus	AY605544	AY605545	AY605546
Heliconius erato	AY918906	AY918907	AY918907
Heliconius melpomene	AY918897	EU480690	EU480690
Limentitis arthemis astyanax	AY918902	AY918903	AY918903
Lycaena helloides	DQ517946	DQ517949	DQ517940
Lycaena heteronea	DQ517947	DQ517950	DQ517941
Lycaena nivalis	DQ517945	DQ517951	DQ517942
Lycaena rubidus	AY587903	AY587901	
Neominois ridingsii		DQ924377	DQ924377
Oeneis chryxus		DQ924378	DQ924378
Pieris rapae		AB086066	AB086066
Polyommatus icarus	DQ402501	EU088114	EU088115
Satyrium behrii	DQ402498	EU352198	EU449023
Agrotis segetum	KF539429	KF539438	KF539450
Agrotis ypsilon	KF539430	KF539439	KF539451
Argyrogramma agnata	KF539431	KF539440	KF539452
Bombyx mori		NM_001043417	
Chilo supressalis	KF539432	KF539441	KF539453
Helicoverpa armigera	KF539433	KF539442	KF539454
Loxostege sticticalis	KF539434	KF539443	KF539455
Macroglossum stellatarum	KF539426	KF539444	KF539456
Mamestra brassicae	KF539427	KF539445	KF539457
Manduca sexta	AD001674	L78080	L78081
Mythimna separata	KF539428	KF539446	KF539458
Plutella xylostella	KF539435	KF539447	
Spodoptera exigua	KF539436	KF539448	KF539459
Spodoptera litura	KF539437	KF539449	KF539460
